# Clinicopathological features of papillary thyroid carcinoma in HIV-infected patients

**DOI:** 10.3389/fonc.2023.1071923

**Published:** 2023-05-05

**Authors:** Jia Liu, Deqian Wu, Jinxin Zhu, Su Dong

**Affiliations:** ^1^Department of Thyroid Surgery, General Surgery Center, First Hospital of Jilin University, Changchun, China; ^2^Department of Anesthesia, First Hospital of Jilin University, Changchun, China

**Keywords:** papillary thyroid carcinoma, thyroid cancer, clinicopathological features, HIV, infection

## Abstract

**Background:**

Papillary thyroid carcinoma (PTC) is the most common endocrine malignancy, with an increasing incidence over the last decades. Human immunodeficiency virus (HIV)-induced immune deficiency was one of risk factors for cancer tumorigenesis and development. The aim of this study was to describe the clinicopathological features of PTC in HIV-infected patients and discuss possible connections between PTC and HIV infection.

**Methods:**

A total of 17670 patients from September 2009 to April 2022 who underwent PTC surgery for the first time were analyzed retrospectively. At last, 10 patients of PTC with HIV infection (HIV-positive group) and 40 patients without HIV infection (HIV-negative group) were included. The differences in general data and clinicopathological characteristics between the HIV-positive group and the HIV-negative group were analyzed.

**Results:**

There were statistically significant differences in age and gender between the HIV-positive group and the HIV-negative group (*P*<0.05), and males and <55 years old accounted for a higher proportion in the HIV-positive group. The differences in tumor diameter and capsular invasion between the HIV-positive group and HIV-negative group were statistically significant (*P*<0.05). Meanwhile, in terms of extrathyroid extension (ETE), lymph node metastasis and distant metastasis, the HIV-positive group were significantly higher than the HIV-negative group (*P*<0.001).

**Conclusion:**

HIV infection was a risk factor for larger tumors, more severe ETE, more lymph node metastasis, and more distant metastasis. HIV infection could promote PTC proliferation and make PTC more aggressive. Many factors such as tumor immune escape, secondary infection, etc. may are responsible for these effects. More attention and more thorough treatment should be paid to these patients.

## Introduction

Papillary thyroid carcinoma(PTC)is the most common endocrine malignancy, with an increasing incidence over the last decades (1). Usually, distant metastases from PTC are rare and the prognosis of PTC is relatively good (2). However, a few populations are considered to be at high risk of poor prognosis (3). Some factors have been associated with poor pathological features and clinical outcomes of PTC, such as BRAFV600E mutation (4), extrathyroidal invasion (5) and so on.

HIV/AIDS prevalence in China during more than a decade indicate that HIV/AIDS prevalence is getting more and more serious and the rapid spread of HIV exists with the characteristics of regional and age differences (6). Some research revealed that HIV-induced immune deficiency was one of risk factors for cancer tumorigenesis and development (7, 8). Other HIV-associated factors contribute to development of malignancies, including direct effect of HIV on various cellular processes, coinfection with oncogenic organisms, risk behaviors (e.g. alcohol or tobacco use), environmental oncogenic factors and possibly antiretroviral therapy (9).

The incidences of thyroid cancer in HIV-infected patients were 4%-8.5% according to previous reports (8, 10). With increased prevalence of PTC, PTC with concomitant HIV infection has become more common in recent years. It is well known that HIV infection can affect thyroid function (11), regulate the Hypothalamic-Pituitary-Thyroid Axis (12, 13) and cause papillomavirus infection (14). However, whether HIV infection is related to the occurrence and development of PTC and the impact of HIV infection on the clinicopathological characteristics of PTC are still unknown. The objective of this study is to describe the clinicopathological features of PTC in HIV-infected patients and discuss possible connections and effects between PTC and HIV infection.

## Materials and methods

### Patients

Data from a total of 17670 hospitalized patients undergoing PTC surgery for the first time from September 2009 to April 2022 at the first hospital of Jilin University were collected. Patients were enrolled if they met all of the following inclusion criteria (1): the patient had a complete documentation of his present and previous diseases (2); the patient underwent initial surgery treatment and the pathological result of the patient was clearly diagnosed as PTC by pathologists (3); without other pathologic types of thyroid cancer (4); there was no history of other malignant tumors. Tumor burden has been assessed preoperatively using imaging including color doppler ultrasonography and pulmonary CT scans as routine. If metastasis is suspected, Emission Computed Tomography (ECT) and PET/CT have been used to evaluate both the number and size of metastases as well as the number of organs involved. We collected ten cases of papillary thyroid carcinoma with HIV-infection and only one patient admitted that he was received systemic treatment for half a year before surgery. But the left nine patients were all the first time diagnosed HIV infection before surgery during screening of infection marker test and didn’t have any relative clinical features of HIV infection. In order to increase the test power of statistical analyses, we randomly matched HIV-positive group and HIV-negative group according to 1:4.

### Surgery and clinicopathological characteristics

All patients underwent unilateral or total thyroidectomy (TT) with unilateral/bilateral central lymph node dissection (CLND), 1671 patients (9.46%) also received a unilateral/bilateral lateral lymph node dissection (LLND). Intraoperative nerve monitoring was done to evaluate recurrent laryngeal nerve (RLN) function and protect the nerve from disturbance. Thyroid function and several related proteins tests were performed. *BRAF* mutation was detected. The following clinical characteristics of PTC patients were recorded: gender, age at diagnosis of PTC and HIV infection, body mass index (BMI), and family history. The pathological features of PTC were as follows: maximum diameter of the tumor, multifocal lesions, bilateral lesions, capsular invasion only, extrathyroid extension (ETE), lymph node metastasis, distant metastasis, combined with Hashimoto’s thyroiditis (HT), pathological TNM stage (using the American Joint Committee on Cancer 8th edition TNM staging system).

### Statistical analysis

SPSS 24.0 statistical software was used for statistical analysis. Measurement data conforming to a normal distribution were expressed as χ ± SD, and the t-test was used for comparisons between groups. Nonparametric tests were used for rank data and continuous data with a nonnormal distribution. Statistical data were expressed by percentage or number of cases, and comparison between groups was performed by Student’s independent samples t test, χ2 test and Fisher’s exact test. Logistic regression analysis was used for multivariate analysis. All statistical tests were two-sided, and differences were considered statistically significant at a *P* value of less than 0.05.

## Results

### Clinicopathological features of the overall data

A detailed presentation of the clinicopathological features of enrolled patients of PTC can be accessed in **Table 1**. Our cohort comprised 14576 (82.49%) females and 3094 (17.51%) males among the 17670 patients, and the mean age of the patients was 46.7 ± 13.8 years. 3028 patients were > 55 years old (17.14%). The median tumor size was 0.94 ± 0.77 cm, and 12370 patients (70.01%) were papillary thyroid microcarcinoma (PTMC). Multifocal lesions were detected in 7313 cases (41.39%), bilateral lesions in 5583 cases (31.6%), thyroid capsule invasion in 12144 cases (68.73%), ETE in 2255 cases (12.76%), lymph node metastasis (N1a and N1b) in 6574 cases (37.2%), and distant metastasis in 152 cases (0.86%). The median TSH level was 2.02 (range: 0.35–4.94 μIU/mL), the median FT3 level was 4.48 (range: 2.43–6.01 pmol/L), and the median FT4 level was 14.08 (range: 9.01–19.05 pmol/L). BRAF^V600E^ mutation test was performed in 1373 patients, of whom 1101 (80.19%) patients were positive. 10 patients were HIV infected. There were 8022 (45.4%) patients who underwent lobectomy, while the remaining 9648 (54.6%) underwent TT. Lateral neck node dissection for 1591 (9%). The numbers of patients in each TNM stage were as follows: 14454 (81.8%) in stage I, 459 (2.6%) in stage II, 1683 (9.52%) stage III, and 1074 (6.08%) stage IV.

**Table 1:** Clinicopathological features of all patients.

Variables	Total (n)	%
Sex		
Male	3094	17.51
Female	14576	82.49
Age (mean ± SD, y)	46.7±13.8	/
55y	14642	82.83
≥55y	3028	17.14
BMI		
24 kg/m^2^	6768	38.3
≥24 kg/m^2^	10902	61.7
Tumor size (mean ± SD, cm)	0.94±0.77	/
≤1cm	12370	70.01
1cm	5300	29.99
Multifocality	7313	41.39
Bilaterality	5583	31.6
Capsular invasion only	12144	68.73
Extrathyroidal extension	2255	12.76
Lymph node metastasis		
N1a	6303	35.67
N1b	1619	9.16
Distant metastasis	152	0.86
Thyroid parameters (mean ± SD)		
TSH (μIU/mL)	2.02±4.01	/
FT3 (pmol/L)	4.48±2.37	/
FT4 (pmol/L)	14.08±10.22	/
BRAF^V600E^ mutation (+/all)	1101/1373	80.19
Hashimoto’s thyroiditis	5315	30.08
HIV infection (+/all)	10/21227	0.047
Surgical intervention		
Total thyroidectomy	9648	54.6
Lobectomy	8022	45.4
AJCC TNM Stage		
I /II	14454/459	81.8/2.6
III/IV	1683/1074	9.52/6.08

### Comparison of clinicpathological features between the HIV-positive and HIV-negative groups

**Figure 1:**
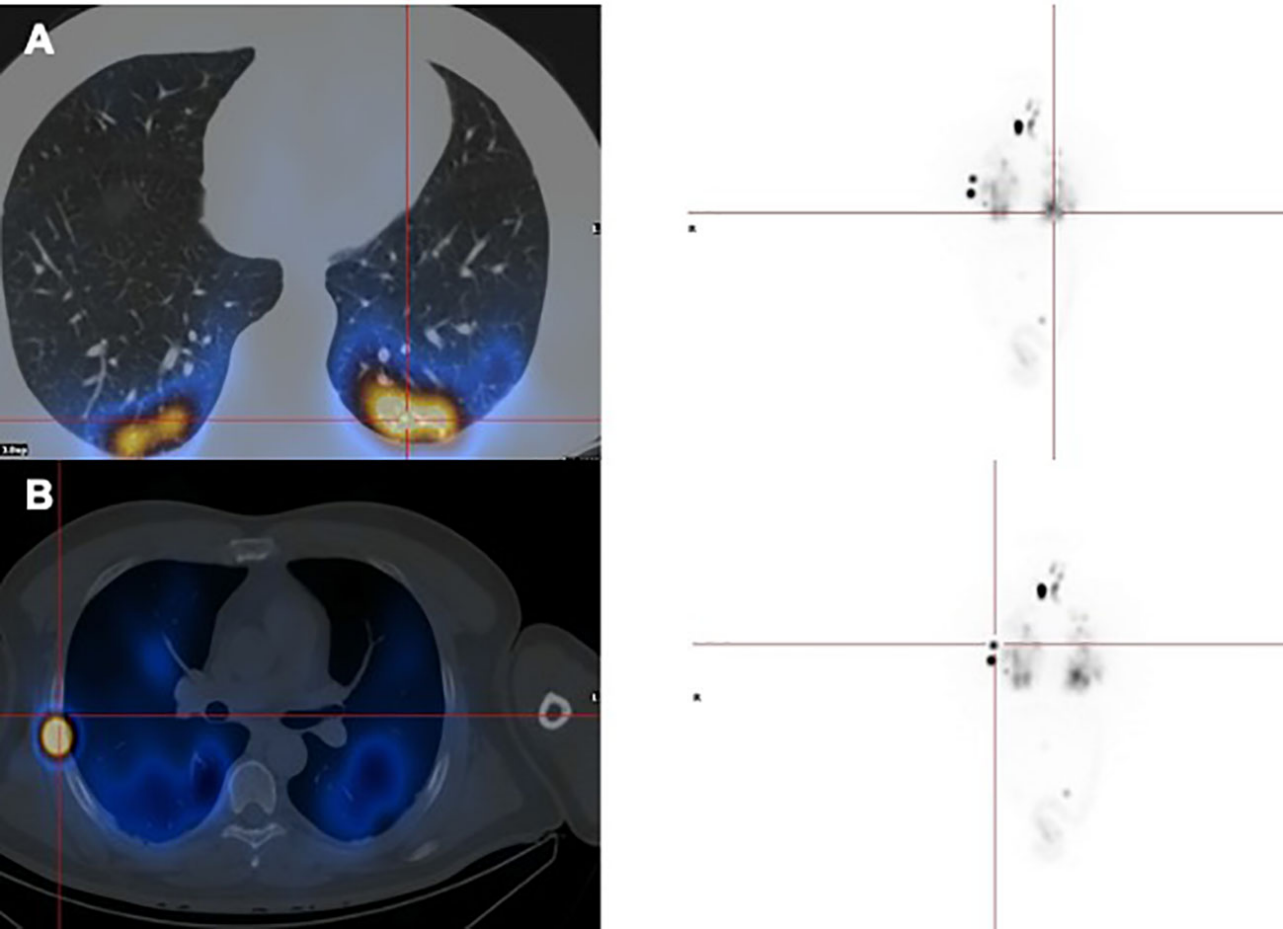
Distant metastasis of PTC in a HIV-infected patient (SPECT/CT). **(A)** Multiple lung metastases, **(B)** Right rib metastasis.

**Figure 2:**
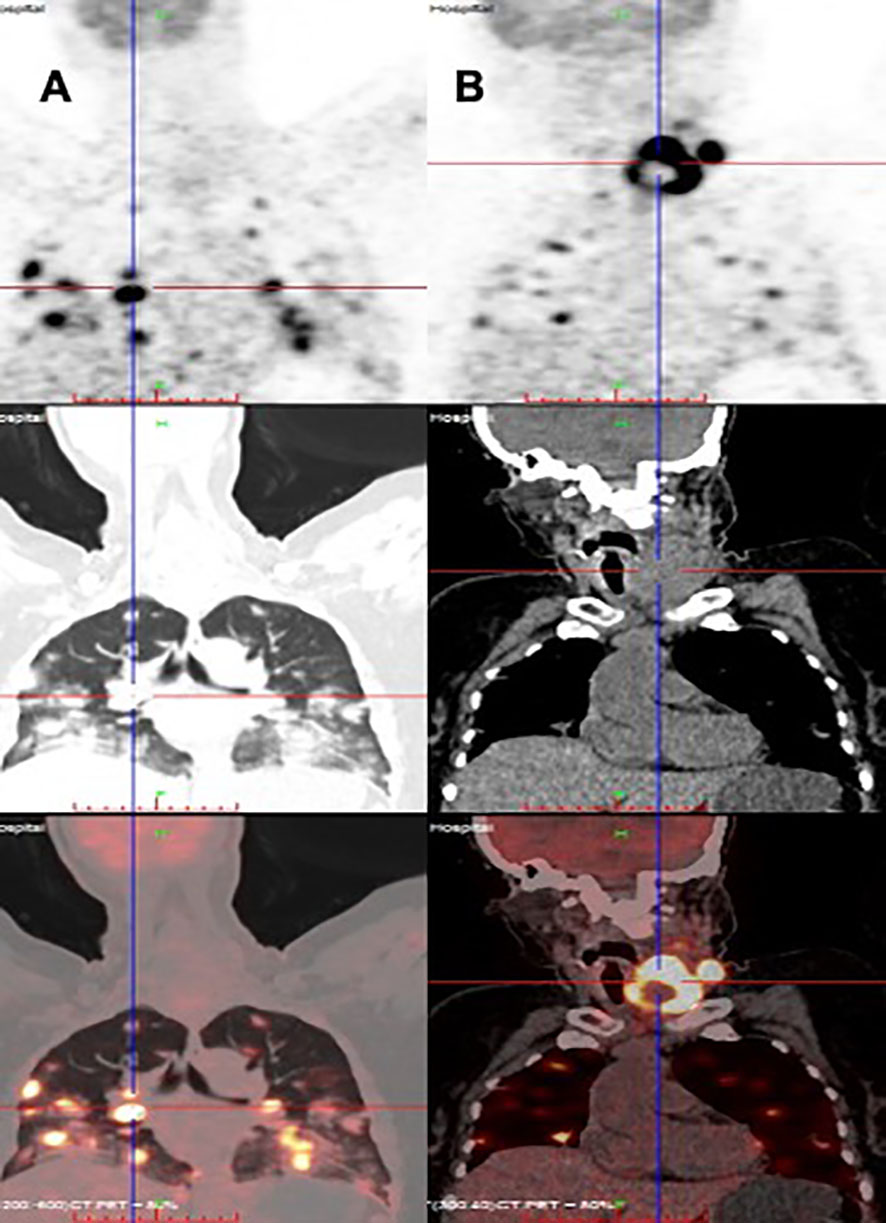
Distant metastasis of PTC in a HIV-infected patient (PET/CT). **(A)** Multiple lung metastases, **(B)** papillary thyroid carcinoma.

There were statistically significant differences in age and gender between the HIV-positive group and the HIV-negative group (*P*<0.05), and males and <55 years old accounted for a higher proportion in the HIV-positive group. The HIV-positive group had a lower BMI than the HIV-negative group, though differences were not statistically significant. The differences in tumor diameter and capsular invasion between the HIV-positive group and HIV-negative group were statistically significant (*P*<0.05). Meanwhile, in terms of ETE, the HIV-positive group was significantly higher than the HIV-negative group (*P*<0.001). In terms of lymph node metastasis and distant metastasis, the proportions of the HIV-positive group were higher than those of the HIV-negative group, differences were statistically significant (*P*<0.001) (**Table 2**, **Figures 1**, **2**). In addition, there were no significant differences in Hashimoto’s thyroiditis, multifocal lesions, bilateral lesions, BRAF^V600E^ mutation, TSH, FT3 and FT4 between patients with and without HIV infection (**Table 2**).

**Table 2:** Comparison of clinicopathological features between HIV-positive and HIV-negative groups.

Variables	HIV (+) (n=10)	HIV (-) (n=40)	t/χ2	*p* value
Sex				
Male	8 (80%)	7 (17.5%)	12.234	<0.001
Female	2 (20%)	33 (82.5%)		
Age (mean ± SD, y)	28.7±7.9	46.7±13.7	6.694	0.012
55y	10 (100%%)	33 (82. 5%)		
≥55y	0 (0%)	7 (17. 5%)		
BMI				
24 kg/m^2^	5 (50%)	16 (40%)	4.152	0.053
≥24 kg/m^2^	5 (50%)	24 (60%)		
Tumor size (mean ± SD, cm)	2.03±1.32	0.95±0.76	11.454	<0.001
≤1cm	2 (20%)	28 (70%)		
1cm	8 (80%	12 (30%)		
Multifocality	5 (50%)	16 (40%)	3.732	0.099
Bilaterality	4 (40%)	13 (32.5%)	2.921	0.124
Capsular invasion only	9 (90%)	27 (67.5%)	5.821	0.017
Extrathyroidal extension	6 (60%)	5(12.5%)	31.301	<0.001
Lymph node metastasis				
N1a	9 (90%)	14 (35%)	47.988	<0.001
N1b	7 (70%)	4 (10%)	65.321	<0.001
Distant metastasis	4 (40%)	1 (2.5%)	80.002	<0.001
Thyroid parameters (mean ± SD)				
TSH (μIU/mL)	3.97±3.83	2.02±4.00	1.363	0.088
FT3 (pmol/L)	2.68±2.11	4.48±2.37	3.559	0.057
FT4 (pmol/L)	13.85±9.62	14.09±10.22	0.572	0.401
BRAF^V600E^ mutation (+/all)	8 (80%)/10	32 (80%)/40	0.037	0.836
Hashimoto’s thyroiditis	4 (40%)	12 (30%)	0.281	0.9

### Association between HIV infection and clinicopathological features of PTC

Univariate and multivariate analyses were performed to detect the correlation between clinicopathological features of PTC and HIV infection. As shown in **Table 3**, in univariate analysis, larger tumor (OR=4.78, 95% CI:2.51–8.94), more severe capsular invasion (OR=1.6, 95% CI:1.07–2.42) and ETE (OR=14.67, 95% CI:7.12–30.13), more lymph node metastasis (N1a: OR=18.24, 95% CI:8.81–33.92; N1b: OR=19.02, 95% CI:4.72–76.54), and more distant metastasis (OR=3.29, 95% CI:1.98–5.77) were associated with HIV infection. Furthermore, after correction for age, sex, TSH, FT3, FT4 and BMI, HIV infection was still a risk factor for larger tumors (OR=5.05, 95% CI:1.86–3.92), more severe ETE (OR=4.12, 95% CI:1.88–9.98), more lymph node metastasis (N1a: OR=5.18, 95% CI:2.66–12.08; N1b: OR=9.56, 95% CI:4.04–22.64), and more distant metastasis (OR=7.16, 95% CI:2.32–20.18).

**Table 3:** Association between HIV infection and clinicopathological features of PTC.

Variables	Univariate analysis			Multivariate analysis		
	OR	95% CI	*p*	OR	95% CI	*p*
Tumor size	4.78	2.51~ 8.94	<0.001	5.05	1.86~3.92	0.01
Capsular invasion only	1.6	1.07~2.42	0.032	1.56	0.90~2.66	0.12
Extrathyroidal extension	14.67	7.12~30.13	<0.001	4.12	1.88~9.98	<0.001
Lymph node metastasis						
N1a	18.24	8.81~33.92	<0.001	5.18	2.66~12.08	<0.001
N1b	19.02	4.72~76.54	<0.001	9.56	4.04~22.64	<0.001
Distant metastasis	3.29	1.98~5.77	<0.001	7.16	2.32~20.18	<0.001

OR, Odds ratio; CI, Confidence interval.

## Discussion

HIV infection is associated with dysfunction of many endocrine organs and their axis, including thyroid gland (15, 16). Various types of thyroid disorders have been described in HIV-infected patients, such as Graves’ disease, hypothyroidism, euthyroid sick syndrome, etc (17–19). Meanwhile, some researches revealed that HIV-induced immune deficiency was the most common risk factor to develop malignancies (8–10).

During more than a decade, AIDS-defining cancers remain frequent in China, and non-AIDS-defining cancers play an important role in morbidity and mortality of HIV positive population in China (8). However, less is known about the impact of HIV infection on PTC so far. Here, we investigate the clinicopathological features of PTC in patients with HIV and discuss possible connections between PTC and HIV infection.

In this study, age and gender distribution had a significant difference in both groups. There were more males and the patients were younger in HIV-positive group. It was consistent with the background of the increasing proportion of young homosexual men in China in recent years. These people have been the fastest-growing risk group for HIV epidemic (20, 21). Moreover, the impact of HIV infection on life expectancy is another reason for the younger age of HIV-positive patients (22). Some researchers believed that HIV infection leads to altered metabolism, poor oral intake and increased prevalence of weight loss (23, 24). BMI was used as a major measurement in most studies. Epidemiology studies have suggested that higher BMI was associated with an increased risk of PTC (25). However, the BMI of the HIV-positive group was slightly lower than that of the HIV-negative group according to our data, but the difference was not significant. This means that HIV infection is unlikely to affect PTC through changes in BMI.

We found that the tumor was larger in the HIV-positive group. It suggested HIV infection could promote PTC proliferation. In addition to, HIV infection also makes PTC more aggressive. Capsular invasion, ETE, lymph node metastasis, and distant metastasis were significantly more common in HIV-infected PTC patients. Several possible reasons are as follows (**Table 4**) (1). Numerous studies have confirmed that hypothroidism and mildly elevated TSH level were more frequently observed in HIV-positive population as compared to HIV-negative group (26–29). In our study, mean TSH value was indeed higher in the HIV-infected patients than in the control group, although the difference was not statistically significant. This agrees with previous findings. TSH is released from the anterior pituitary under positive regulation from TSH-releasing hormone (which is released from the hypothalamus) and negative feedback from the thyroid hormones tri-iodothyronine (T3) and thyroxine (T4). HIV infection frequently results in early and protracted disturbances of hypothalamus/pituitary and thyroid dysfunction, which could change TSH concentrations in serum (30). Higher TSH has a proliferative effect on PTC growth that is most likely mediated by TSH receptors on tumor cells (31) (2); The immune system is no longer functioning effectively after HIV infection, which enable cancer cells to escape from immune surveillance and develop rapidly (32, 33) (3); Secondary virus and carcinogen infections are common in patients with HIV. For example, some viruses, such as human papillomavirus, cytomegalovirus, Epstein-Barr virus, herpes virus, etc., promote tumorigenesis and development of PTC (34–36) (4); Genetic factors may play roles in the pathogenesis of PTC in HIV-positive patients. Yahong Chen et al. had identified nearly 40 HIV-related genes from widely biological pathways (37). Molecular biology has discovered several pathways that play major roles in the occurrence and development of PTC. For example, *VHL* is a well-known tumor suppressor that is deregulated in the majority of PTC tissues (38). *STAT1* promotes cell proliferation and inhibits apoptosis via AMPK signaling pathway in PTC (39). *NCOR2* accelerates PTC progression by upregulating metastasis-associated protein 2 expression (40) (5); Highly active anti-retroviral therapy (HAART) may alter clinicopathological features of PTC by drug interactions or effects on the immune system (41) (6); Cachexia, psychological effects and stress with HIV infection are also the important clinical risk factors of PTC (42) (7); Changes of thyroid-related cytokines after HIV infection, such as thyroxine-binding globulin (TBG), rT3 and anti-TPO antibody may also play roles. But further studies are needed here (43, 44) (8); Direct cytopathic effects of HIV on the thyroid gland. Here we could put forward such hypotheses: HIV can affect the normal physiological status of the thyroid gland by altering CD4 and CD8 (45, 46), or HIV affects thyroid hormone metabolism through the interaction with peripheral T3 receptors, and then affects the status of thyroid follicular cells (47).

**Table 4:** Potential factors of HIV influencing PTC.

Hypothalamic-pituitary-thyroid axis	
Tumor immune escape
Secondary virus and carcinogen infections
Genetic factors
Drugs
Cachexia, psychological effects and stress
Changes of thyroid-related cytokines
Direct cytopathic effects of HIV

As a preliminary study, there are still some limitations in this study. Firstly, the sample size of the study is relatively small in HIV-positive group. We only collected 10 patients of PTC with HIV infection (HIV-positive group) from total of 17670 patients who underwent PTC surgery for the first time during the past 13 years in our department. Perhaps the reasons for the great difference are the lower incidence of HIV infection and higher incidence of thyroid deceases in northeast of China. Secondly, there is only one patient who received anti-virus therapy for a short time before surgery and we can’t get more favorable information to compare the different clinicopathological features of papillary thyroid carcinoma of performing HIV therapy or not. The potential biases of the study may because more diagnosed HIV (+) patients were transferred to hospital for infectious diseases to performing surgery and systematic anti-virus therapy, which result in less cases collected. Thirdly, Serum-based biomarkers, such as lactate dehydrogenase, can also reflect tumor burden and are often also correlated with a poor response to immune-checkpoint inhibitors. Other circulating markers (such as circulating free tumor DNA and/or circulating tumor cells) are also attracting research interest as surrogate markers of tumor burden. Herein, we didn’t compare the serum-based biomarkers and hope more complete data can be collected and analyzed to enrich the clinicopathological features in the follow-up study.

## Conclusion

Our study described that the clinicopathological features of PTC in patients with HIV. HIV infection could promote PTC proliferation and make PTC more aggressive. Many factors such as tumor immune escape, secondary infection, etc. may are responsible for these effects. More attention and more thorough treatment should be paid to these patients.

## Data availability statement

The raw data supporting the conclusions of this article will be made available by the authors, without undue reservation.

## Ethics statement

The studies involving human participants were reviewed and approved by ethics committee of first hospital of jilin university. Written informed consent for participation was not required for this study in accordance with the national legislation and the institutional requirements.

## Author contributions

JL wrote the article. JZ and DW collected data. SD analyzed data and gave administration support. All authors listed have made a substantial, direct, and intellectual contribution to the work and approved it for publication. All authors contributed to the article and approved the submitted version.

## References

[1] Borazan H Kececioglu A Okesli S Otelcioglu S. Oral magnesium lozenge reduces postoperative sore throat: a randomized, prospective, placebo-controlled study. Anesthesiology (2012) 117(3):512–8. doi: 10.1097/ALN.0b013e3182639d5f 22797283

[2] Zhang C Li B Zhang L Chen F Zhang Y Cheng W. Clinicopathological and ultrasound features as risk stratification predictors of clinical and pathological nodal status in papillary thyroid carcinoma: a study of 748 patients. BMC Cancer (2022) 22(1):354. doi: 10.1186/s12885-022-09474-8 35365120 PMC8976313

[3] So YK Kim MJ Kim S Son YI. Lateral lymph node metastasis in papillary thyroid carcinoma: a systematic review and meta-analysis for prevalence, risk factors, and location. Int J Surg (2018) 50:94–103. doi: 10.1016/j.ijsu.2017.12.029 29329789

[4] Yan C Huang M Li X Wang T Ling R. Relationship between BRAF V600E and clinical features in papillary thyroid carcinoma. Endocr Connect (2019) 8(7):988–96. doi: 10.1530/EC-19-0246 PMC6652244 31252408

[5] Zhao J Zhang Y Zheng X. Clinicopathological characteristics of papillary thyroid cancer located in the isthmus with delphian lymph node metastasis. Br J Oral Maxillofac Surg (2021) 60(5):635–638. doi: 10.1016/j.bjoms.2021.11.016 35210104

[6] Qiao YC Xu Y Jiang DX Wang X Wang F Yang J. Epidemiological analyses of regional and age differences of HIV/AIDS prevalence in China, 2004-2016. Int J Infect Dis (2019) 81:215–20. doi: 10.1016/j.ijid.2019.02.016 30797071

[7] Grulich AE van Leeuwen MT Falster MO Vajdic CM. Incidence of cancers in people with HIV/AIDS compared with immunosuppressed transplant recipients: a meta-analysis. Lancet (2007) 370(9581):59–67. doi: 10.1016/S0140-6736(07)61050-2 17617273

[8] Wang F Xiang P Zhao H Gao G Yang D Xiao J. A retrospective study of distribution of HIV associated malignancies among inpatients from 2007 to 2020 in China. Sci Rep (2021) 11(1):24353. doi: 10.1038/s41598-021-03672-3 34934097 PMC8692320

[9] Proulx J Ghaly M Park IW Borgmann K. HIV-1-Mediated acceleration of oncovirus-related non-AIDS-Defining cancers. Biomedicines (2022) 10(4):768. doi: 10.3390/biomedicines10040768 35453518 PMC9024568

[10] Basilio-De-Oliveira CA. Infectious and neoplastic disorders of the thyroid in AIDS patients: an autopsy study. Braz J Infect Dis (2000) 4(2):67–75. 10795071

[11] Dev N Sahoo R Kulshreshtha B Gadpayle AK Sharma SC. Prevalence of thyroid dysfunction and its correlation with CD4 count in newly-diagnosed HIV-positive adults–a cross-sectional study. Int J STD AIDS (2015) 26(13):965–70. doi: 10.1177/0956462414563776 25505045

[12] Pekic S Miljic D Popovic V Feingold KR Anawalt B Blackman MR. Infections of the hypothalamic-pituitary region. In: Feingold KR Anawalt B Boyce A Chrousos G de Herder WW Dhatariya K, editors. South Dartmouth (MA): Endotext [Internet]. MDText.com, Inc.; (2000).

[13] Merenich JA. Hypothalamic and pituitary function in AIDS. Baillieres Clin Endocrinol Metab (1994) 8(4):757–67. doi: 10.1016/S0950-351X(05)80298-8 7811219

[14] Dona MG Di Bonito P Chiantore MV Amici C Accardi L. Targeting human papillomavirus-associated cancer by oncoprotein-specific recombinant antibodies. Int J Mol Sci (2021) 22(17):9143. doi: 10.3390/ijms22179143 34502053 PMC8431386

[15] Zandman-Goddard G Shoenfeld Y. HIV And autoimmunity. Autoimmun Rev (2002) 1(6):329–37. doi: 10.1016/S1568-9972(02)00086-1 12848988

[16] Pommier JD Laouenan C Michard F Papot E Urios P Boutten A. Metabolic syndrome and endocrine status in HIV-infected transwomen. AIDS (2019) 33(5):855–65. doi: 10.1097/QAD.0000000000002152 30664006

[17] Parsa AA Bhangoo A. HIV And thyroid dysfunction. Rev Endocr Metab Disord (2013) 14(2):127–31. doi: 10.1007/s11154-013-9248-6 23743889

[18] Ugwueze CV Young EE Unachukwu CN Onyenekwe BM Nwatu CB Okafor CI. The prevalence and pattern of thyroid dysfunction in HAART-naive HIV patients in enugu, Nigeria: a cross-sectional comparative study. West Afr J Med (2021) 38(12):1200–5. doi: 10.55891/wajm.v38i12.49 35037450

[19] Jariyawattanarat V Sungkanuparph S Sriphrapradang C. Characteristics of graves disease in hiv-infected patients on antiretroviral therapy. Endocr Pract (2020) 26(6):612–8. doi: 10.4158/EP-2019-0514 31968184

[20] Delaugerre C Gallien S Flandre P Mathez D Amarsy R Ferret S. Impact of low-level-viremia on HIV-1 drug-resistance evolution among antiretroviral treated-patients. PloS One (2012) 7(5):e36673. doi: 10.1371/journal.pone.0036673 22590588 PMC3349708

[21] Xu J Luo Y Dong H Zhao G. The effects of Internet exposure on sexual risk behavior among sexually experienced Male college students in China: cross-sectional study. JMIR Public Health Surveill (2022) 8(5):e31847. doi: 10.2196/31847 35499864 PMC9112083

[22] Morales DR Moreno-Martos D Matin N McGettigan P. Health conditions in adults with HIV compared with the general population: a population-based cross-sectional analysis. EClinicalMedicine (2022) 47:101392. doi: 10.1016/j.eclinm.2022.101392 35497059 PMC9046106

[23] Harmooshi NN Abeshtan A Zakerkish M Mirmomeni G Rahim F. The effect of metformin on body mass index and metabolic parameters in non-diabetic HIV-positive patients: a meta-analysis. J Diabetes Metab Disord (2021) 20(2):1901–11. doi: 10.1007/s40200-021-00869-1 PMC8630165 34900832

[24] Njoku PO Ejim EC Anisiuba BC Ike SO Onwubere BJ. Clinical and echocardiographic findings in a cross-sectional study of HIV-infected adults in enugu, Nigeria. Cardiovasc J Afr (2021) 32(6):320–6. 10.5830/CVJA-2020-065 PMC8756073 34128946

[25] Zhao S Jia X Fan X Zhao L Pang P Wang Y. Association of obesity with the clinicopathological features of thyroid cancer in a large, operative population: a retrospective case-control study. Med (Baltimore) (2019) 98(50):e18213. doi: 10.1097/MD.0000000000018213 PMC6922396 31852078

[26] Thaimuta ZL Sekadde-Kigondu C Makawiti DW. Thyroid function among HIV/AIDS patients on highly active anti-retroviral therapy. East Afr Med J (2010) 87(12):474–80. 23457856

[27] Amadi K Sabo AM Ogunkeye OO Oluwole FS. Thyroid hormone: a "prime suspect" in human immunodeficiency virus (HIV/AIDS) patients? Niger J Physiol Sci (2008) 23(1-2):61–6. doi: 10.4314/njps.v23i1-2.54927 19434216

[28] Wang JJ Zhou JJ Yuan XL Li CY Sheng H Su B. Hyperthyroidism caused by acquired immune deficiency syndrome. Eur Rev Med Pharmacol Sci (2014) 18(6):875–9. 24706313

[29] Otieno CF. Thyroid dysfunction in HIV/AIDS–cause or consequence? East Afr Med J (2010) 87(12):473. 23457855

[30] Collazos J Ibarra S Mayo J. Thyroid hormones in HIV-infected patients in the highly active antiretroviral therapy era: evidence of an interrelation between the thyroid axis and the immune system. AIDS (2003) 17(5):763–5. doi: 10.1097/00002030-200303280-00019 12646804

[31] Demircioglu ZG Demircioglu MK Aygun N Akgun IE Unlu MT Kostek M. Relationship between thyroid-stimulating hormone level and aggressive pathological features of papillary thyroid cancer. Sisli Etfal Hastan Tip Bul (2022) 56(1):126–31. doi: 10.14744/SEMB.2022.14554 PMC9040308 35515965

[32] Shindiapina P Ahmed EH Mozhenkova A Abebe T Baiocchi RA. Immunology of EBV-related lymphoproliferative disease in HIV-positive individuals. Front Oncol (2020) 10:1723. doi: 10.3389/fonc.2020.01723 33102204 PMC7556212

[33] Castle PE Burk RD Massad LS Eltoum IE Hall CB Hessol NA. Epidemiological evidence that common HPV types may be common because of their ability to evade immune surveillance: results from the women's interagency HIV study. Int J Cancer (2020) 146(12):3320–8. doi: 10.1002/ijc.32693 PMC7373334 31577842

[34] Ortiz-Gutierrez F Sanchez-Minutti L Martinez-Herrera JF Torres-Escobar ID Pezzat-Said EB Marquez-Dominguez L. Identification of genetic variants of human papillomavirus in a group of Mexican HIV/AIDS patients and their possible association with cervical cancer. Pol J Microbiol (2021) 70(4):501–9. doi: 10.33073/pjm-2021-047 PMC8702602 35003280

[35] Gernert M Kiesel M Frohlich M Renner R Strunz PP Portegys J. High prevalence of genital human papillomavirus infection in patients with primary immunodeficiencies. Front Immunol (2021) 12:789345. doi: 10.3389/fimmu.2021.789345 34868076 PMC8637119

[36] Pereira LMS Franca EDS Costa IB Lima IT Freire ABC Ramos FLP. Epstein-Barr Virus (EBV) genotypes associated with the immunopathological profile of people living with HIV-1: immunological aspects of primary EBV infection. Viruses (2022) 14(2):168. doi: 10.3390/v14020168 35215762 PMC8880155

[37] Chen Y Yuan J Han X Liu X Han X Ye H. Coexpression analysis of transcriptome on AIDS and other human disease pathways by canonical correlation analysis. Int J Genomics (2017) 2017:9163719. doi: 10.1155/2017/9163719 28695125 PMC5488239

[38] Baldini E Tuccilli C Arlot-Bonnemains Y Chesnel F Sorrenti S De Vito C. Deregulated expression of VHL mRNA variants in papillary thyroid cancer. Mol Cell Endocrinol (2017) 443:121–7. doi: 10.1016/j.mce.2017.01.019 28089820

[39] Li HP Yang TZ Wei L Ge YF Meng QS. STAT1-induced upregulation of lncRNA LINP1 promotes cell proliferation and inhibits apoptosis via AMPK signaling pathway in papillary thyroid cancer. Eur Rev Med Pharmacol Sci (2020) 24(17):8911–7. 10.26355/eurrev_202009_22832 32964981

[40] Luan S Fu P Wang X Gao Y Shi K Guo Y. Circular RNA circ-NCOR2 accelerates papillary thyroid cancer progression by sponging miR-516a-5p to upregulate metastasis-associated protein 2 expression. J Int Med Res (2020) 48(9):300060520934659. doi: 10.1177/0300060520934659 32940102 PMC7503031

[41] Marima R Hull R Lolas G Syrigos KN Kgoebane-Maseko M Kaufmann AM. The catastrophic HPV/HIV dual viral oncogenomics in concert with dysregulated alternative splicing in cervical cancer. Int J Mol Sci (2021) 22(18):10115. doi: 10.3390/ijms221810115 34576278 PMC8472041

[42] Possel P Mitchell AM Harbison B Fernandez-Botran GR. Association of cancer caregiver stress and negative attribution style with depressive symptoms and cortisol: a cross-sectional study. Support Care Cancer (2022) 30(6):4945–52. doi: 10.1007/s00520-022-06866-1 PMC8854472 35179652

[43] Hommes MJ Romijn JA Endert E Adriaanse R Brabant G Eeftinck Schattenkerk JK. Hypothyroid-like regulation of the pituitary-thyroid axis in stable human immunodeficiency virus infection. Metabolism (1993) 42(5):556–61. doi: 10.1016/0026-0495(93)90212-7 8492709

[44] Sharma N Sharma LK Dutta D Gadpayle AK Anand A Gaurav K. Prevalence and predictors of thyroid dysfunction in patients with HIV infection and acquired immunodeficiency syndrome: an Indian perspective. J Thyroid Res (2015) 2015:517173. doi: 10.1155/2015/517173 26798547 PMC4700191

[45] Akinsete A Oyenusi E Odugbemi B Odugbemi T Temiye E. Spectrum of thyroid abnormalities among children living with HIV in Lagos, Nigeria. J Thyroid Res (2019) 2019:1096739. doi: 10.1155/2019/1096739 31015954 PMC6448344

[46] Emokpae MA Akinnuoye IM. Asymptomatic thyroid dysfunction in human immunodeficiency virus-1-infected subjects. J Lab Physicians (2018) 10(2):130–4. doi: 10.4103/JLP.JLP_172_16 PMC5896176 29692575

[47] Hsia SC Wang H Shi YB. Involvement of chromatin and histone acetylation in the regulation of HIV-LTR by thyroid hormone receptor. Cell Res (2001) 11(1):8–16. doi: 10.1038/sj.cr.7290061 11305329

